# Extreme sensitivity of reservoir computing to small network disruptions

**DOI:** 10.1186/1471-2202-16-S1-P256

**Published:** 2015-12-18

**Authors:** Philippe Vincent-Lamarre, Guillaume Lajoie, Jean-Philippe Thivierge

**Affiliations:** 1 School of Psychology and Center for Neural Dynamics, University of Ottawa, Ottawa, Ontario K1N 6N5, Canada; 2 UW Institute for Neuroengineering, University of Washington, Seattle, WA, US; 3 Max Planck Institute (DS) and Bernstein Center for Computational Neuroscience, Göttingen, Germany

## 

**Figure 1:**
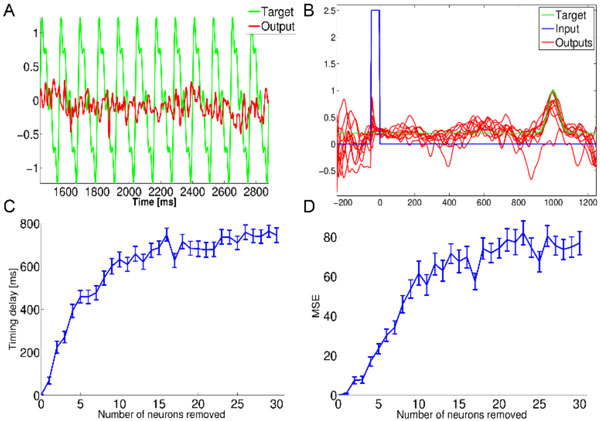
**Performance of damaged reservoirs of 1,000 neurons with FORCE and innate learning algorithms**. **A**. Target signal (green, perfectly replicated with the originally trained network) and the trace of the same network after the removal of one neuron in its reservoir. **B**. Ten trials (red) with different initial conditions of a damaged network (N-2 neurons) that is trained to peak at 1,000 ms (green) using innate learning. **C**. Average lag between the target and the output timing (100 trials per condition) as a function of the number of removed neurons. **D**. Mean squared error as a function of the number of removed neurons.

Recent computational models based on reservoir computing (RC) are gaining attention as plausible theories of cortical information processing. In these models, the activity of a recurrently connected population of neurons is sent to one or many read-out units through a linear transformation. These models can operate in a chaotic regime which has been proposed as a possible mechanism underlying sustained irregular activity observed in cortical areas [1,2]. Furthermore, models based on RC replicate the neural dynamics involved in decision making [3], interval timing [2], and motor control [1]. However, one biological constraint that has been overlooked in these models is their resistance to small connectivity perturbations such as failures in synaptic transmission, a phenomenon that occurs frequently in healthy circuits without causing any drastic functional changes. Here, we show that different implementations of RC display very little resistance to small synaptic disruptions and discuss the implications of such fragility for RC mechanisms that may be present in neural coding. With the FORCE [1] procedure, networks lost their ability to replicate a jagged sinusoidal signal after a single neuron was removed from the reservoir (Figure 1A). Networks with innate training [2] showed a similar effect on a timing task (Figure 1B). The lag in the timing and the noise in the output both increased monotonically as further neurons were removed (Figure 1C,D); networks reached random performance after ~1.5% of neurons were eliminated. After the suppression of a single neuron, the spectrum of the weight matrix was greatly disturbed and repeated trials displayed unreliable trajectories, as assessed with principal components analysis. When individual synapses were removed instead of neurons, networks reached random performance after ~0.5% of synapses from the reservoir were eliminated. While living neuronal circuits can withstand small synaptic disruptions without compromising task performance, our results suggest that such disruptions have a catastrophic impact on the behaviour of RC models. Retraining the read-out unit seems to be futile as it results as a completely new solution post retraining instead of a finer restructuration. These results cast doubt on the validity of a large class of models that claim to capture the neuronal mechanisms of cognitive and behavioral tasks.
